# Rapid evolution of insecticide resistance and patterns of pesticides usage in agriculture in the city of Yaoundé, Cameroon

**DOI:** 10.1186/s13071-022-05321-8

**Published:** 2022-06-02

**Authors:** Nadège Sonhafouo-Chiana, Leslie Diane Nkahe, Edmond Kopya, Parfait Herman Awono-Ambene, Samuel Wanji, Charles Sinclair Wondji, Christophe Antonio-Nkondjio

**Affiliations:** 1 Organisation de Coordination pour la lutte Contre les Endémies en Afrique Centrale (OCEAC), Institut de Recherche de Yaoundé (IRY), P.O. Box 288, Yaoundé, Cameroon; 2 Parasites and Vector Research Unit (PAVRU), Department of Microbiology and Parasitology, University of Buea, P.O. Box 63, Buea, Cameroon; 3 Faculty of Science, University of Yaoundé I, P.O. Box 337, Yaoundé, Cameroon; 4 Centre for Research in Infectious Diseases (CRID), P.O. BOX 13591, Yaoundé, Cameroon; 5 Research Foundation in Tropical Diseases and Environment (REFOTDE), P.O. Box 474, Buea, Cameroon; 6 Vector Biology, Liverpool School of Tropical medicine, Pembroke Place, Liverpool, L3 5QA UK

**Keywords:** Vector control, *Anopheles gambiae*, Insecticide resistance, Pesticide management, Yaoundé, Cameroon

## Abstract

**Background:**

The practice of agriculture in urban settings contributes to the rapid expansion of insecticide resistance in malaria vectors. However, there is still not enough information on pesticide usage in most urban settings. The present study aims to assess the evolution of *Anopheles gambiae* (s.l.) population susceptibility to insecticides and patterns of pesticide usage in agriculture in the city of Yaoundé, Cameroon.

**Methods:**

WHO susceptibility tests and synergist PBO bioassays were conducted on adult *An. gambiae* (s.l.) mosquitoes aged 3 to 5 days emerging from larvae collected from the field. Seven insecticides (deltamethrin, permethrin, DDT, bendiocarb, propoxur, fenitrothion and malathion) were evaluated. The presence of target site mutation conferring knockdown (*kdr*) resistance was investigated using TaqMan assay, and mosquito species were identified using SINE-PCR. Surveys on 81 retailers and 232 farmers were conducted to assess general knowledge and practices regarding agricultural pesticide usage.

**Results:**

High resistance intensity to pyrethroids was observed with a high frequency of the *kdr* allele 1014F and low frequency of the *kdr* 1014S allele. The level of susceptibility of *An. gambiae* (s.l.) to pyrethroids and carbamates was found to decrease with time (from > 34% in 2017 to < 23% in 2019 for deltamethrin and permethrin and from 97% in 2017 to < 86% in 2019 for bendiocarb). Both *An. gambiae* (s.s.) and *An. coluzzii* were recorded. Over 150 pesticides and fertilizers were sold by retailers for agricultural purposes in the city of Yaoundé. Most farmers do not respect safety practices. Poor practices including extensive and inappropriate application of pesticides as well as poor management of perished pesticides and empty pesticide containers were also documented.

**Conclusions:**

The study indicated rapid evolution of insecticide resistance and uncontrolled usage of pesticides by farmers in agriculture. There is an urgent need to address these gaps to improve the management of insecticide resistance.

**Graphical Abstract:**

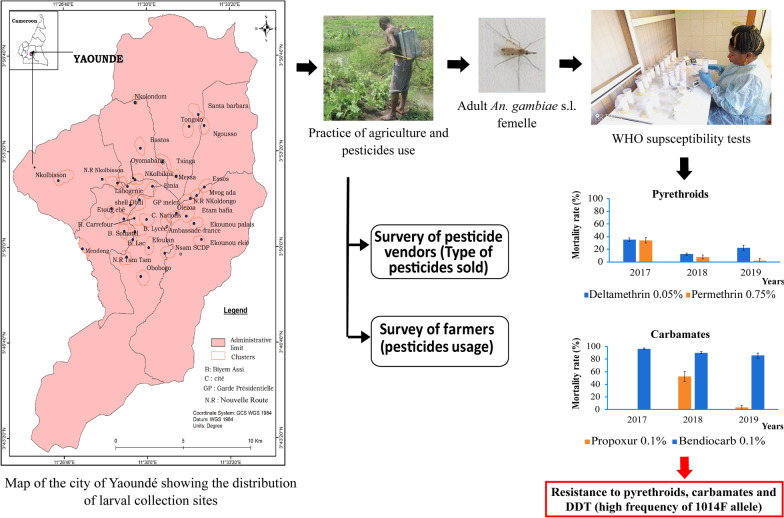

**Supplementary Information:**

The online version contains supplementary material available at 10.1186/s13071-022-05321-8.

## Background

Despite progress in malaria control registered during the last decade, following the large-scale deployment of insecticide-based interventions such as long-lasting insecticidal nets (LLINs) and indoor residual spraying (IRS) [1, 2], malaria remains an important public health problem in Africa [3, 4]. In Cameroon, malaria is still a major threat. Prevention strategies rely mainly on the use of LLINs. Over 35 million LLINs have been distributed across the country so far. It is estimated that over 70% of households own at least a net and that 58% of the population uses nets regularly [5, 6]. However, the sustainability of these insecticide-based control interventions is challenged by the spread of insecticide resistance in main malaria vectors [7–9]. Studies conducted across the country reported increased expansion of insecticide resistance in the major malaria vectors *An. gambiae* (s.l.) and *An. funestus* [7, 10–13].

Pesticides used in agriculture are considered to largely contribute to the selection and spread of insecticide resistance in *An. gambiae* (s.l.) [10, 14–18]. Mosquitoes are now becoming increasingly tolerant to several compounds including pyrethroids, organochlorines, carbamates and organophosphates.

Pyrethroids are the only insecticides approved by WHO for impregnating mosquito nets [19] because of their low toxicity to humans and other mammals, quick knockdown effect and cost-effectiveness [20, 21]. The other insecticides (organophosphates, carbamates and organochlorines) are mainly used for indoor residual spraying [19]. One of the mechanisms involved in pyrethroid resistance in *An. gambiae* (s.l.) is target-site insensitivity, also known as knockdown resistance (*kdr*), induced by two different mutations occurring at position 1014 on the voltage-gated sodium channel gene (*VGSC*). The first mutation leads to a leucine-to-phenylalanine substitution and is widely distributed in West Africa [22], whereas the second leads to a leucine-to-serine substitution and is largely expanded in East Africa [23]. In addition to target site mechanisms, resistance could also occur through overexpression of detoxification enzymes. The overexpression of P450 genes has been found associated with resistance to organochlorines, pyrethroids and carbamates [11, 24–26]. Overexpression of glutathione-S transferase genes, notably the *GSTe2* gene, is associated with DDT resistance [27]. *GST*s are more active in *An. funestus* and have been associated to many cases of resistance to both pyrethroids and carbamates [12, 28]. The current evolution of insecticide resistance in vector population calls for urgent actions to improve control.

Selections by insecticide use in public health and agriculture are all considered to drive the rapid expansion of insecticide resistance in malaria vectors [29]. However, so far there have been few investigations on pesticide usage in relation with insecticide resistance expansion [30]. The last decade has shown increasing demand for pesticides with countries such as China, the USA and Argentina accounting for 70% of global pesticides used in agriculture (2.44 billion kg of active ingredient annually) [31]. Cameroon is one of the 13 countries which consume between 10 to 50 million kg of pesticides in agriculture. The utilization of pesticides in Cameroon has increased eightfold during the last decade, and the current increase is in line with the extension of agricultural land surfaces [31]. It is possible that the quantity of pesticides used could be underestimated since many pesticides and fertilizers used in the country elude controls at the borders [32–34]. Although there are effective laws guiding pesticide and fertilizer supply, selling and utilization, this regulation is not always applied [35, 36]. A large variety of pesticides are sold in local markets or on the street. These pesticides of unknown quality could expose the population to hazards and affect pest and vector-borne disease control [37–40]. In the present study, an assessment of the evolution of insecticide resistance in vector populations was conducted alongside a survey on pesticides sold and used by farmers in the city of Yaoundé.

## Methods

### Sampling site and mosquito collection

**Fig. 1:**
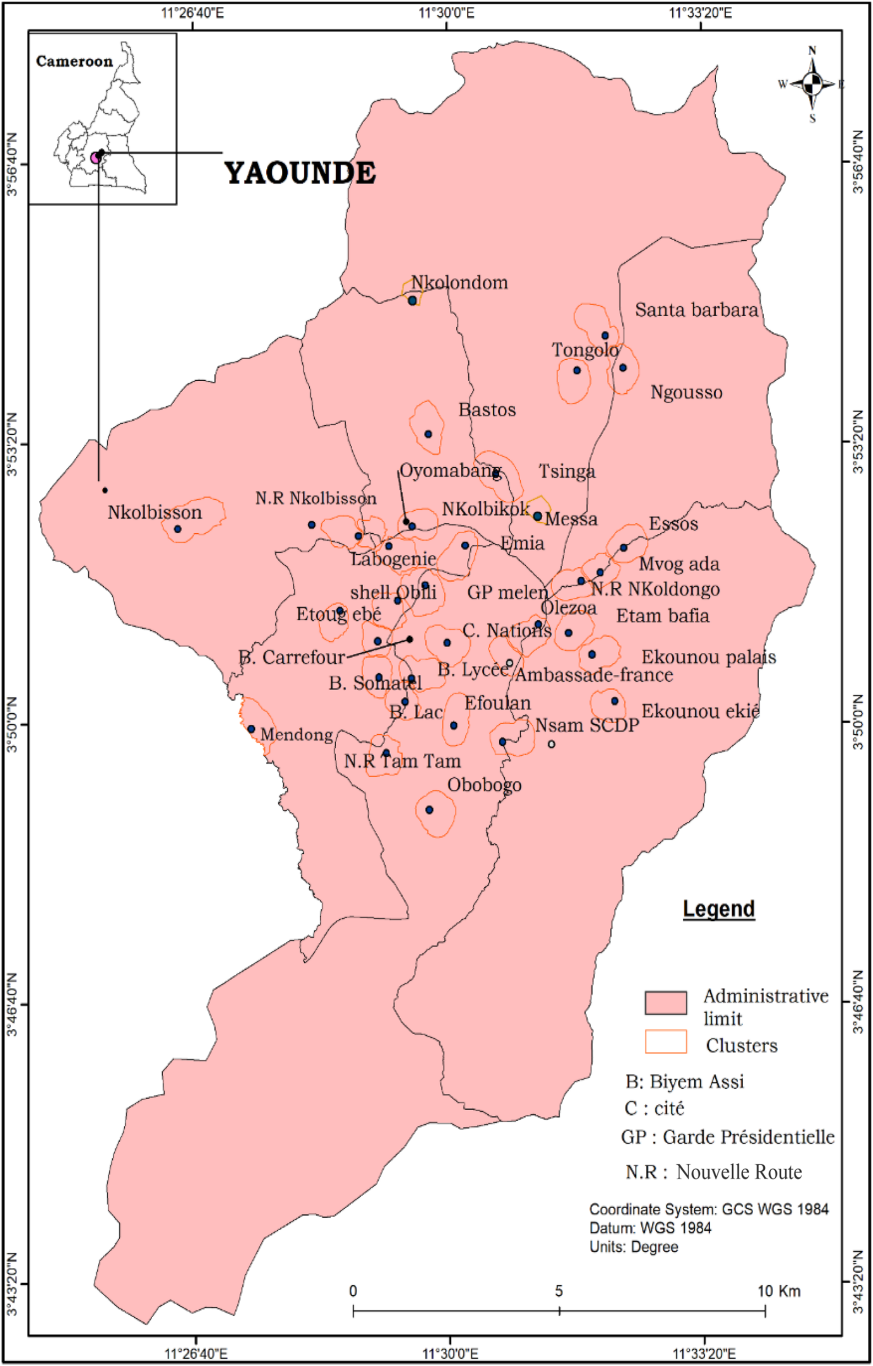
A map of the city of Yaoundé showing the distribution of larval collection sites [the map of Yaoundé is available in open access on the OpenStreetMap platform (https://www.openstreetmap.org/search?query=cameroon#map=6/7.406/12.283)]

Mosquito larval collections were conducted in 32 districts of Yaoundé (Fig. 1), the capital city of Cameroon (3˚52′N; 11˚31′E). Yaoundé is situated within the Congo-Guinean phytogeographic domain and has an equatorial climate consisting of four seasons: two rainy seasons (March–June and September–November; annual rainfall 1700 mm) and two dry seasons (December–February and July–August). Yaoundé's landscape comprises high and low land areas. Low land areas include large swamps, lakes and rivers and are frequently exploited for market gardening. The practice of market gardening has now largely expanded in the city centre particularly along the edges of rivers. More than ten rivers cross the city, the most important being Mfoundi, Mefou and Biyeme Rivers. The periphery of vast Yaoundé zones has been deforested, and these areas are exploited for agriculture. Crops cultivated by inhabitants include maize, vegetables, groundnuts and beans. About 10% of the city population practices agriculture on different scales.

### Larval collections and mosquito rearing

The immature stages of *An. gambiae* (s.l.) were collected using the standard dipping technique [41]. The latter consists of collecting mosquito larvae from the surface of the breeding sites using a ladle. Larval collections were conducted in standing water collections present in cultivated agricultural areas and different places across the city. Water collections were sometimes clean or full of organic matter. These water collections were mainly found in lowland areas or close to swamps. After collection, larvae were kept in labelled jars according to surveyed sites and then transported to the insectary at OCEAC (Organization of Coordination of the Fight against Endemic Diseases in Central Africa) for rearing. Larvae were raised under standard temperature (27 °C ± 2) and humidity (65% ± 10) conditions until the adult stage. Adult anophelines were identified to the species level using morphological identification keys [42, 43].

### Insecticide susceptibility bioassays

Bioassays were carried out using the standard WHO protocol [44]. Tests were performed with WHO-supplied insecticide-impregnated papers. Insecticides tested included two pyrethroids at different doses (0.05% deltamethrin, 0.25% deltamethrin, 0.5% deltamethrin; 0.75% permethrin, 3.75% permethrin and 7.5% permethrin), one organochlorine (4% DDT), two carbamates (0.1% bendiocarb and 0.1% propoxur) and two organophosphates (1% fenitrothion and 5% malathion). Three- to 5-day-old unfed female *An. gambiae* (s.l.) collected at the larval stage and reared until adult stage were exposed for 1 h to these insecticides. Mosquitoes were divided into batches of 25 individuals before being exposed to insecticide-treated papers for 1 h. Experiments were conducted at a temperature of 22 to 26 °C with a minimum of four replicates per bioassay, and the mortality rates were recorded after 24 h. The insecticide-susceptible strains of *An. gambiae* (s.l.) (Kisumu and Ngousso strains) were used as control to assess the quality of the impregnated papers. For control tests, silicone-treated papers were used. The result of the insecticide susceptibility test was valid if mortality in the control group was < 5% and discarded if the mortality in the control was > 20%. When the mortality rate was between 5 and 20%, the mortality rate was corrected using Abbott’s formula [45]. For each insecticide, dead mosquitoes were kept separately in 1.5-ml microtubes containing silica gel, whereas mosquitoes still alive after the tests and control samples were kept separately in RNAlater tubes for molecular analysis.

### Synergist bioassay with piperonyl butoxide (PBO)

Following the high level of resistance recorded against 0.75% permethrin and 0.05% deltamethrin, the effect of the synergist PBO in combination with these insecticides was tested to assess the potential contribution of P450 monooxygenase enzymes. Subsamples of 20 to 25 unfed, 3–5-day-old adult females of *An. gambiae* (s.l.) randomly collected from a cage were pre-exposed to 4% PBO paper for 1 h before being immediately exposed to 0.75% permethrin or 0.05% deltamethrin for an additional 1 h. Mortality following exposure to both PBO and permethrin or deltamethrin was recorded after 24 h. Susceptibility tests were conducted alongside controls.

### DNA extraction, species identification and detection of *kdr* mutations

Sub-samples of surviving female mosquitoes (alive after 24 h exposure), dead and control mosquitoes were randomly selected for molecular analysis. The Livak method [46] was used to extract genomic DNA from single mosquitoes. Mosquito identification to the species level was carried out using SINE200 PCR for *An. gambiae* (s.l.) [47]. Target site mutations (L1014F and L1014S) in the voltage-gated sodium channel gene of *An. gambiae* (s.l.) mosquitoes were genotyped using TaqMan assay protocol, previously described by Bass et al. [48].

### Knowledge, Attitudes and Practices (KAP) surveys of agrochemical vendors and farmers

KAP surveys of agrochemical vendors and farmers in the city of Yaoundé were conducted using a semi-structured questionnaire (Additional file 1: Table S1). The questionnaire was divided into three sections. The first section collected socio-demographic information on vendors and farmers (age, sex, education level). The second section, designed for the vendors, collected information on pesticides sold, origin of pesticides and interaction between vendors and the end-users. The third part, designed for the farmers, explored the usage of pesticides by farmers, type of pesticide used, frequency of pesticide usage, type of crops cultivated, size of the land cultivated, knowledge of pesticide used, respect of standard dosage, frequency of application and respect of safety measures (protection measures, manipulation of pesticides, management of empty containers, expired pesticides).

### Statistical analysis

Results of WHO susceptibility bioassays and synergist were recorded in Microsoft Excel files and analysed according to WHO criteria [44]. A mosquito population was considered susceptible if the mortality rate was ≥ 98%; when the mortality rate was between 90 and 97% the population was considered possibly resistant but this needed to be checked; when the mortality rate was < 90% the population was considered fully resistant. A test was deemed valid if mortality in the control group was < 5% and discarded if the mortality in the control was > 20% [44]. When the mortality rate of control was between 5 and 20%, the mortality rate of mosquitoes exposed was corrected using Abbott’s formula [45]. The presence of *kdr* alleles was detected using TaqMan qPCR according to Bass et al. [48]. The *kdr* allele frequency was calculated as follows: f(R) = (2 × RR + RS)/2 N and f(S) = 1−f(R), with RR = total number of homozygote resistant, RS = total number of heterozygote resistant and N = total number of mosquitoes successfully screened for the *kdr* mutation. Mosquito populations were checked to determine whether they were in Hardy-Weinberg equilibrium. Data generated through the KAP surveys were analysed in Microsoft Excel. Data cleaning was performed to check for inconsistencies in data entry and responses. Data were analysed using SPSS version 20 statistical software package. Means, frequencies and proportions were used for descriptive analysis of the data. Percentages were compared using chi-squared test. Comparison between means was assessed using ANOVA. The 95% confidence interval (95% CI) was computed using MedCalc v14.8.1 software. Statistical significance was set at *P* < 0.05.

## Results

### Species identification

*Anopheles gambiae* (s.l.) samples were composed of two species: *An. coluzzii* and *An. gambiae* (s.s.). *Anopheles coluzzii* was the most abundant species (Table 1). No major changes in the composition of the anopheline fauna was observed during the 3 years of monitoring (*P* ˃ 0.2).

**Table 1:** Distribution of species of *An. gambiae* (s.l.) complex during surveys in the city of Yaoundé

Species	Years					
	2017		2018		2019	
	n/N	% [95% CI]	n/N	% [95% CI]	n/N	% [95% CI]
*An. coluzzii*	47/52	90.4% [82; 98.8]	293/331	88.52% [84.9; 92.2]	240/254	94.5% [91.6; 97.4]
*An. gambiae* (s.s.)	5/52	9.6% [− 16.2; 35.5]	38/331	11.48% [1.3; 21.6]	14/254	5.5% [− 6.4; 17.5]

*n* number of specimens identified to the species level, *N* total number of specimens processed, *95% CI* 95% confidence interval

### Insecticide resistance profile

**Fig. 2:**
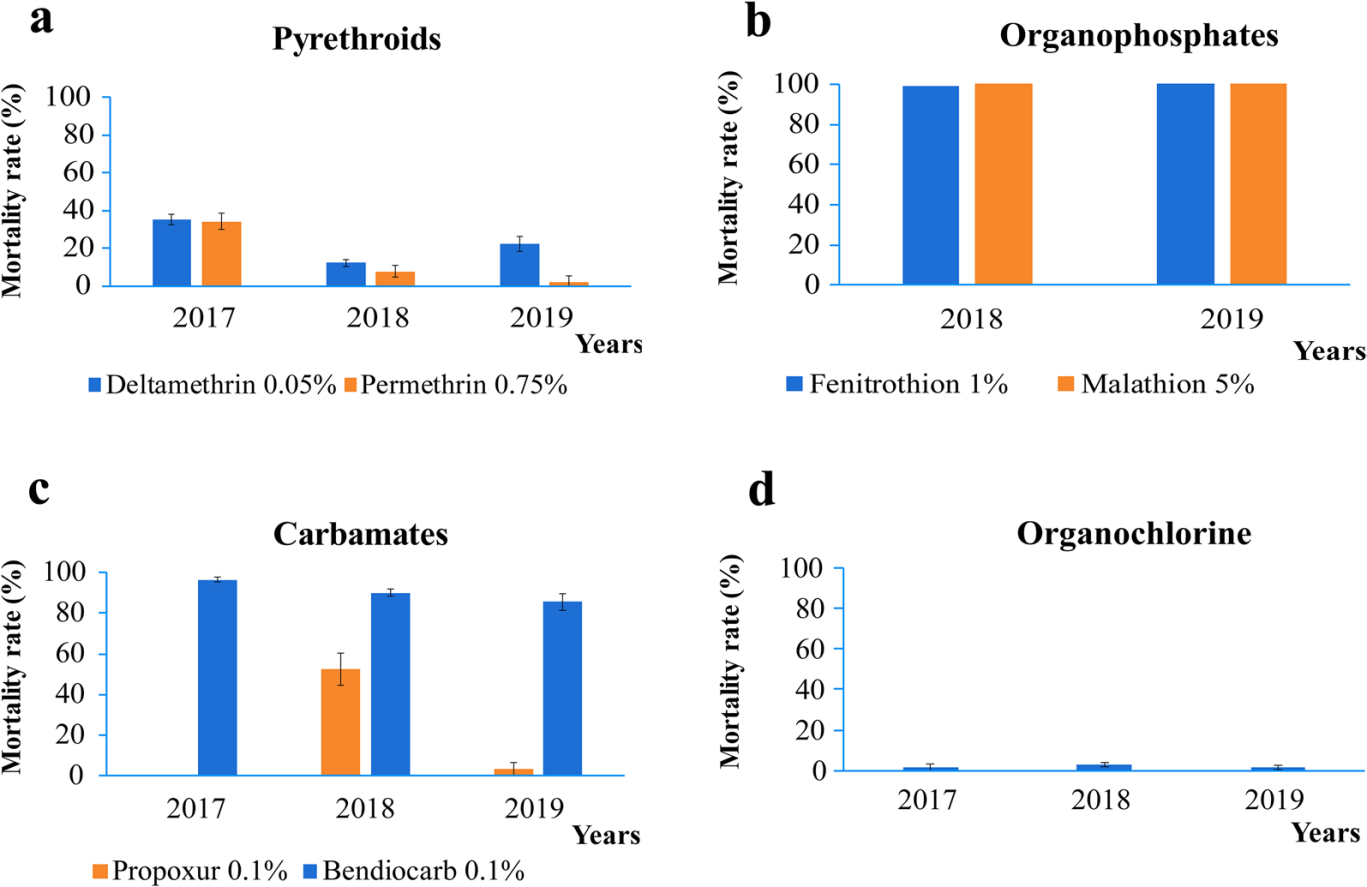
Evolution of *Anopheles gambiae* (s.l.) resistance to pyrethroids (**a** 0.75% permethrin and 0.05% deltamethrin); organophosphates (**b** 1% fenitrothion and 5% malathion); carbamates (**c** 0.1% bendiocarb and 0.1% propoxur); organochlorine (**d** 4% DDT) insecticides in the city of Yaoundé. The error bars represent 95% confidence interval (CI)

A total of 11,894 female *An. gambiae* (s.l.) mosquitoes obtained from larvae and pupae collected in 34 districts in the city of Yaoundé were tested to assess their susceptibility profile to seven insecticides (Fig. 2). Females were chosen because they are the ones transmitting malaria and coming into contact with humans. Mortality rates to deltamethrin varied from 35.2 ± 2.7%, 12.3 ± 1.6% and 22.4 ± 3.9%, respectively, for 2017, 2018 and 2019 (Fig. 2a). Mortality to permethrin, significantly decreased with time from 34.2 ± 4.2% in 2017, 7.7 ± 2.5% in 2018 to 2.1 ± 1.3% in 2019. For the organochlorine DDT, mortality rates were 1.3 ± 1.8%, 3.1 ± 1% and 1.5 ± 1.3% in 2017, 2018 and 2019, respectively (Fig. 2d).

The average mortality rates to bendiocarb varied from 96.4 ± 1.4% in 2017, 90.1 ± 1.7% in 2018 and 85.7 ± 4.1% in 2019 (c). Mortality rate to propoxur was 52.3 ± 7.9% in 2018 and 3.3 ± 3.2% in 2019.

Mortality rates to the organophosphates fenitrothion and malathion were always > 98% in both 2018 and 2019 (b).

### Insecticide resistance intensity to permethrin and deltamethrin

**Fig. 3:**
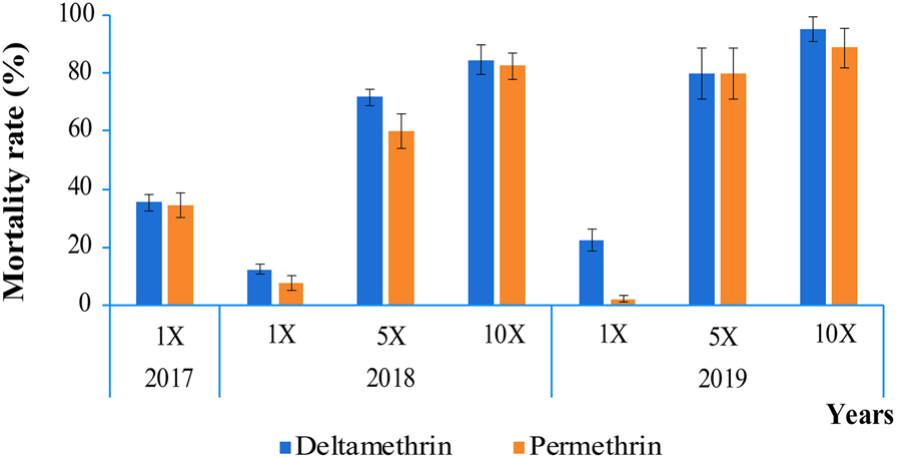
Resistance intensity to permethrin and deltamethrin of *An. gambiae* (s.l.) from the city of Yaoundé. (1× deltamethrin = 0.05% deltamethrin; 1× permethrin = 0.75% permethrin; 5× deltamethrin = 0.25% deltamethrin; 5× permethrin = 3.75% permethrin; 10× deltamethrin = 0.5% deltamethrin; 10× permethrin = 7.5% permethrin). The error bars represent 95% confidence interval (CI)

The mortality rate of *An. gambiae* (s.l.) was found to increase with the concentration of deltamethrin and permethrin (Fig. 3). For deltamethrin and permethrin 5×, the mortality rate varied respectively from 71.7 ± 2.9% and 59.9 ± 6.1% in 2018 to 80 ± 8.8% in 2019. In the case of deltamethrin 10×, the mortality rate varied from 84.6 ± 5.2% in 2018 to 95 ± 4.3% in 2019. For permethrin 10×, the mortality rate varied from 82.8 ± 4.7% in 2018 to 88.8 ± 6.9% in 2019.

### Tests with PBO as synergist

**Fig. 4:**
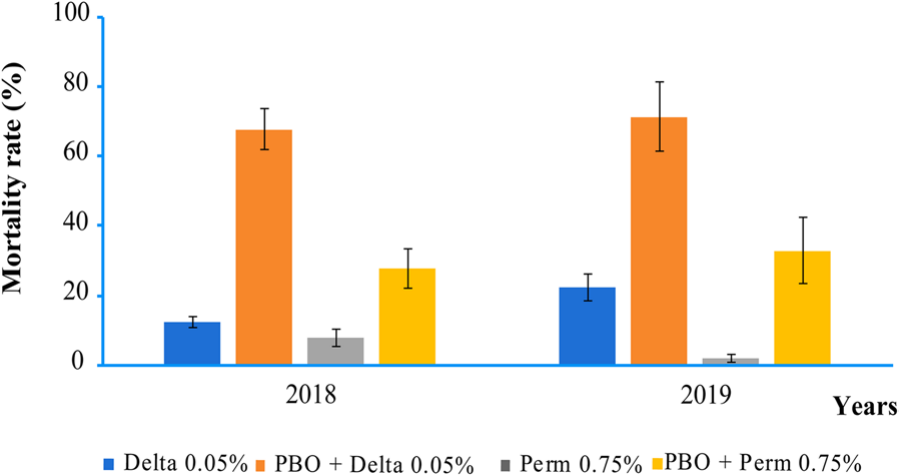
Evolution of pyrethroid resistance in *An. gambiae* (s.l.) when pre-exposed to PBO in 2018 and 2019. (Delta, deltamethrin; PBO, piperonyl butoxide; Perm, permethrin). The error bars represent 95% confidence interval (CI)

Pre-exposure of *An. gambiae* (s.l.) populations to 4% PBO synergist significantly increased the insecticidal activity of both deltamethrin and permethrin. Mortality shifted from 12.3 ± 1.6% for deltamethrin alone to 67.6 ± 5.8% for deltamethrin + PBO in 2018 and from 22.4 ± 3.9% for deltamethrin alone to 71.3 ± 9.9% mortality after pre-exposure to PBO in 2019 (Fig. 4). Similarly, mortality shifted from 7.7 ± 2.5% for permethrin alone to 27.8 ± 5.7% for permethrin + PBO in 2018 and from 2.1 ± 1.3% for permethrin alone to 33 ± 9.7% mortality after pre-exposure to PBO in 2019 (Fig. 4).

### L1014F *kdr* detection in *An. gambiae* (s.s.) and *An. coluzzii*

A total of 801 samples were genotyped between 2017 and 2019 to assess the presence of L1014F *kdr* allele. Of these samples, 43.8% (351/801) were homozygote resistant RR, 53.3% (*n* = 427/801) were heterozygote RS, and only 2.9% (*n* = 23/801) were homozygote susceptible SS. The frequency of the resistant allele 1014F was high and varied from 0.6 to 0.8 (Table 2). None of the samples collected in 2017, 2018 and 2019 appeared to be in Hardy-Weinberg equilibrium (*P* < 0.001).

**Table 2:** Evolution of *kdr* allele L1014F genotypes and allele frequencies in *An. gambiae* (s.l.) populations from the city of Yaoundé

Years	Genotypes						Alleles
	RR		RS		SS		
	*n*/*N*	% [95% CI]	*n/N*	% [95% CI]	*n/N*	% [95% CI]	f(R)
2017	15/68	22.1% [21.9; 22.3]	51/68	75% [74.9; 75.1]	2/68	2.9% [2.7; 3.2]	0.6
2018	221/426	51.9% [51.9; 52]	193/426	45.3% [45.2; 45.4]	12/426	2.8% [2.7; 2.9]	0.8
2019	115/307	37.5% [37.4; 37.6]	183/307	59.6% [59.5; 59.7]	9/307	2.9% [2.8; 3]	0.7

*S* wild type, *R* 1014F, *SS* susceptible homozygote, *RR* resistant homozygote, *RS* resistant heterozygote, *F(R)* frequency of the *Kdr* L1014F allele, *n* number of specimens with *kdr* allele, *N* total number of specimens processed, *95% CI* 95% confidence interval

### L1014S *kdr* detection in *An. gambiae* (s.s.) and *An. coluzzii*

Out of 525 samples genotyped, 2.3% (*n* = 12/525) were heterozygote RS; no homozygote resistant RR was found. The frequency of the resistant allele 1014S was 0.01 in 2017 and 2018. The allele was not present in 2019 samples (Table 3).

**Table 3:** Evolution of *kdr* allele L1014S genotypes and frequencies in *An. gambiae* (s.l.) populations in the city of Yaoundé

Years	Genotypes				Alleles
	RS		SS		
	*n/N*	% [95% CI]	*n/N*	% [95% CI]	f(R)
2017	2/68	2.9% [− 20.5; 26.4]	66/68	97.06% [93; 101.1]	0.01
2018	10/341	2.9% [− 7.5; 13.4]	331/341	97.07% [95.3; 98.9]	0.01
2019	0/116	0%	116/116	100%	0

*S* wild type, *R* 1014S, *SS* susceptible homozygote, *RR* resistant homozygote, *RS* resistant heterozygote, *F(R)* frequency of the *Kdr* L1014S allele, *n* number of specimens with *kdr* allele, *N* total number of specimens processed *95% CI* 95% confidence interval

### Socio-demographic characteristics of pesticide vendors

A total of 81 agrochemical shops were visited in 10 different markets of the city of Yaoundé Nfoundi (*n* = 26), Mokolo (*n* = 12), Vogt Mbi (*n* = 8), Kouabang (*n* = 7), Ekounou (*n* = 6), Mendong (*n* = 5), Vogt Ada (*n* = 5), Etoudi (*n* = 5), Essos (*n* = 4) and Acacias (*n* = 3). General information on the respondents is outlined in Table 4. Most of the sellers [39.5% (*n* = 32/81)] were between 31 and 40 years old. The majority of pesticide vendors had the secondary education level [42% (*n* = 34/81)]. Some had attended the university [39.5% (*n* = 32/81)]; some had never attended school [16.1% (*n* = 13/81)].

**Table 4:** Socio-demographic characteristics of pesticides sellers

Variable	Category	Percentage (*n*)
Gender	Males	48.2% (42)
	Females	51.9% (39)
Age (years)	≤ 30	16.1% (13)
	31–40	39.5% (32)
	41–50	23.5% (19)
	> 50	13.6% (11)
	No answer	7.4% (6)
Education level	Primary school	2.5% (2)
	Secondary school	42% (34)
	University	39.5% (32)
	Illiterate	16.1% (13)

### Pesticides sold on the market

The agricultural pesticides found on the market in Yaoundé included: insecticides, fungicides, herbicides, nematicides and acaricides (Additional file 2: Table S2). These compounds were used for different purposes: plant protection against pests, home weeding and indoor spraying. Pesticides were sold alongside fertilizers.

### Active ingredients, chemical classes and application doses

Different classes of compounds including pyrethroids, organophosphates, organochlorines, carbamates, nicotinoids and neonicotinoids were commonly found in pesticides sold on the market (Additional file 2: Table S2). Details on the composition, active ingredient, dilution and application dose were always provided. Almost all pesticides had broad-spectrum activity and acted by killing pests either after ingestion or through direct contact or inhalation. Most pesticides were multi-site inhibitors affecting various enzymes and metabolic processes in pests. Many pesticides contained a mixture of two active ingredients at different doses. Active ingredients commonly found in each insecticide family were: pyrethroids (e.g. deltamethrin, cypermethrin, lambda-cyhalothrin, bifenthrin) or organophosphates (e.g. chlorpyrifos, chlorpyrifos-thyl, pirimiphos methyl), carbamates (e.g. mancozeb, oxamyl, maneb), organochlorines (e.g. chlorothalonil), neonicotinoids (e.g. imidacloprid) and nicotinoids (e.g. acetamiprid). Other active ingredients included fungicides (e.g. mancozeb, metalaxyl-M, dimethomorph, chlorothalonil, mefenoxam, cuprous oxide, carbendazim, cymoxanil). Glyphosate, paraquat, nicosulfuron, 2,4-D amine salt, fluroxypy, triclopyr butoxyethyl ester and triclopyr acid equivalent were the main herbicides recorded and oxamyl and abamectin the main nematicide. Insecticide formulations commonly found on the market were emulsifiable concentrate (EC) and suspension concentrate (SC). Most herbicides and fungicides were formulated as soluble concentrate (SL) and wettable powders (WP), respectively.

### WHO toxicity classification

Most of the insecticides found during the study (Additional file 2: Table S2) belonged to WHO Class II (moderately toxic or hazardous or dangerous); others were slightly hazardous insecticides (WHO Class III) and nocif or harmful (Class Xn). Most fungicides were classified as slightly hazardous (WHO Class III). Most herbicides were slightly hazardous insecticides (WHO Class III) and unlikely dangerous in normal use (WHO Class U). Highly hazardous pesticides (WHO Class Ib) were found in herbicides (Additional file 2: Table S2).

### Pesticides sold and sources

Insecticides were the most common pesticide (48%, *n* = 72/150), followed by fungicides (26%, *n* = 39/150) and herbicides (23.3%, *n* = 35/150) (Additional file 2: Table S2 and Table 5). Nematicides and acaricides accounted for 2.7% (*n* = 4/150). Most products originated from Asia (86.7%, *n* = 130/150). The remaining products (13.3%, *n* = 20/150) originated from Europe, America and Africa. Pesticides sold were mainly used in agriculture (96.7%, *n* = 145/150) while just a few (3.3%, *n* = 5/150) were used for indoor spraying. The demand for pesticides was high (93.8%, *n* = 76/81) between March and June during the short rainy season at the beginning of agricultural activities.

**Table 5:** Different selling practices reported by pesticide vendors during the survey

Variable	Answer	Percentage response (*n*)
Type of pesticide sold	Insecticides	48% (72)
	Fungicides	26% (39)
	Herbicides	23.3% (35)
	Nematicides and acaricides	2.7% (4)
Origin of pesticides sold	Asia	86.7% (130)
	Europe	8.7% (13)
	Africa	2.7% (4)
	America	2% (3)
Usage of pesticides and fertilizers	Agriculture	96.7% (145)
	Indoor spraying	3.3% (5)
Purchasing period	March–June (rainy season)	93.8% (76)
	July–August (dry season)	1.2% (1)
	September–October (rainy season)	3.7% (3)
	November–February (dry season)	1.2% (1)
Do you advise customers?	Yes, regularly	100% (81)
	No	0% (0)
Advise provided to customers	Choice of product	70.4% (57)
	Dosage	28.4% (23)
	Personal protection	1.2% (1)
Do pesticides and fertilizers often perish?	Yes	80.6% (65)
	No	19.8% (16)
What do you do with expired pesticides and fertilizers?	Return to suppliers	60% (39)
	Used on the field	26.2% (17)
	Sell them	6.2% (4)
	Throw them in the trash	7.7% (5)

### Pesticide usage and safe practices

All pesticide retailers (100%, *n* = 81/81) reported regularly advising their customers on the safe usage of pesticides (Table 5). Advice provided concerned the choice of the product (70.4%, *n* = 57/81) (based on the type of crops which could be sprayed and pest or crop diseases targeted by the compound), how to prepare the doses for field application (28.4%, *n* = 23/81) and personal protection measures (1.2%, *n* = 1/81). Most retailers (80.3%, *n* = 65/81) reported that pesticides and fertilizers often perished in their shops. Many reported not selling those stocks but returning them to their suppliers (60%, *n* = 39/65). Some admitted selling them (6.2%, *n* = 4/65), using them in the field (26.2%, *n* = 17/65) or throwing them in the trash (7.7%, *n* = 5/65)].

### Socio-demographic characteristics and socio-economic status of farmers interviewed during the survey

The socio-economic and demographic profile of the 232 farmers who participated in the survey is summarized in Table 6. Most of the farmers were male [58.6% (*n* = 136)]. The age range of farmers varied from 20 to > 50 years. Concerning their level of education, 22% (*n* = 51/232) had primary school level, 39.2% (*n* = 91/232) secondary, 34.9% (*n* = 81/232) university level and 3.9% (9/232) no formal education. Most farmers interviewed practiced farming as a part-time activity (79.3%, *n* = 184/232), whereas for 20.7% (*n* = 48/232) of the respondents it was their main activity.

**Table 6:** Socio-demographic characteristics of farmers involved in the survey

Variable	Category	Percentage (*n*)
Gender	Males	58.6% (136)
	Females	41.4% (96)
Age (years)	20	4.7% (11)
	21–30	16.8% (39)
	31–40	31.5% (73)
	41–50	25.9% (60)
	> 50	21.1% (49)
Education level	Primary school	22% (51)
	Secondary school	39.2% (91)
	University	34.9% (81)
	No formal training	3.9% (9)
Occupation	Farmer	20.7% (48)
	Farmer + small scale business	45.3% (105)
	Farmer + civil servant	10.8% (25)
	Unemployed	23.3% (54)

### Sites surveyed

Farmers working in different neighbourhoods of the city of Yaoundé were visited; the areas included: Ekounou ekie, Ahala, Mendong, Nsimeyon, Etoug-ebe, Mokolo, Nkolondom, Ekounou palais, Efoulan, Nsimbock and Nkolbisson. Crops cultivated in the different areas included vegetables, tomatoes, fruit trees, celery, parsley, basil, lettuce, eggplant, leek, chili, cabbage, okra, maize, mint, melon, water melon, cucumber, marzipan, banana, plantain, groundnut, pepper, peanuts, cereals, potatoes, sugar cane, beans, citrus, avocado, cassava and papaya. Pests reported to affect production included a wide range insects (beetles, grasshoppers, flies, ants, bugs, crickets, locusts), scales, worms, biters, suckers, crushers, fungal infections, black pod disease, thrips, caterpillars and aphids.

### Knowledge on pesticide use

Of the 232 farmers interviewed, 87.5% (*n* = 203/232) were small-scale farmers with < 2000 m^2^ land exploited (Table 7). Farmers exploiting land size of 2000 m^2^ to 1 ha represented 11.2% (*n* = 26/232) and farmers exploiting land surface > 1 ha were only 1.3% (*n* = 3/232). Almost all farmers [91% (*n* = 211/232)] reported using both pesticides and fertilizers. Only 9.1% (*n* = 21/232) of the farmers reported not using pesticides simply because of lack of financial means. Among pesticides, those most commonly applied on crops were insecticides. Insecticides were mostly used in combination with other compounds such as fungicides, nematicides, acaricides and herbicides. The proportion of farmers using mixtures of insecticides + fungicides represented 49.8% (*n* = 105/211). The proportion mixing insecticides + fungicides + nematicides + acaricides was 33.65% (*n* = 71/211); those mixing insecticides + fungicides + herbicides was 2.4% (*n* = 5/211). Those using insecticides alone represented 14.2% (*n* = 30/211).

**Table 7:** Different usage practices of pesticides by farmers in Yaoundé

Variable	Answer	% of farmers (*n*)
Land size	< 2000 m^2^	87.5% (203)
	2000 m^2^–1 ha	11.2% (26)
	> 1 ha	1.3% (3)
Do you use pesticides and synthetic fertilizers?	Yes	91% (211)
	No	9.1% (21)
Pesticides used	Insecticide	14.2% (30)
	Insecticide + fungicide	49.8% (105)
	Insecticide + fungicide + nematicide + acaricide	33.7% (71)
	Insecticide + fungicide + herbicide	2.4% (5)
Where did you learn how to use pesticides?	Advice	80.1% (169)
	Training	15.2% (32)
	Label	4.8% (10)
On what basis do you make the dilutions?	Randomly	10% (21)
	Instructions on label	31.8% (67)
	Advice from suppliers and others	51.2% (108)
	Training or seminar	7.1% (15)
When do you use pesticides and fertilizers?	Dry season	23.2% (49)
	All seasons	76.8% (162)
Respect the recommended doses	Yes (standard doses)	4.3% (9)
	No (high doses)	95.7% (202)
What do you do with the empty containers?	Burn	14.2% (30)
	Bury	7.6% (16)
	Throw in the trash	31.3% (66)
	Discard indiscriminately	36% (76)
	Keep for recycling	10.9% (23)
Frequency of pesticide application during plant cultivation	1 time	17.5% (37)
	Several times (up to 6)	82.5% (174)
Are these pesticides effective?	Yes	93.4% (197)
	No	6.6% (14)
Do pesticides and fertilizers often perish?	Yes	27% (57)
	No	70.1% (148)
	No answer	2.8% (6)
What do you do with expired pesticides and fertilizers?	Burn	3.3% (7)
	Bury	15.2% (32)
	Throw away	70.1% (148)
	Use	5.2% (11)
	Return to the suppliers	2.4% (5)
	No answer	3.8% (8)

### Knowledge on pesticide usage and safety practices

From the study, it appeared that farmers normally use far more than the recommended dosage of various pesticides and fertilizers (Table 7). Almost all farmers (95.8%, *n* = 202/211) admitted not respecting the recommended dosages. Most farmers (80.1%, *n* = 169/211) said they took advice from retailers before using pesticides and fertilizers. Some farmers (15.2%, *n* = 32/211) reported to have been trained in how to grow crops and use pesticides while 4.7% (*n* = 10/211) farmers reported using instructions on labels. Concerning pesticide dilution and the indicated rate of application, most farmers (51.2%, *n* = 108/211) said they took advice from pesticide vendors or family members, friends, neighbours or other farmers. Some (31.8%, *n* = 67/211) reported reading the instructions on labels, 10% (*n* = 21/211) stated that they determined the dosage by themselves and 7.1% (*n* = 15/211) stated that they attended training workshops.

### Frequency of pesticide usage and handling

Pesticides, fertilizers and treatments are applied at different times during crop growing (Table 7). Most farmers reported using pesticides in all seasons (76.8%, *n* = 162/211). Some indicated applying pesticides only during the dry season (23.2%, *n* = 49/211). The spraying device used by farmers was a sprayer. Pesticides were always sprayed in combination with fertilizers. The dose of pesticides sprayed depended on the frequencies of spraying, type of crop cultivated, land size, severity of infection and income status. Usually, pesticides were applied several times, up to six times (82.5%, *n* = 174/211), during plant growth. Most farmers (93.4%, *n* = 197/211) reported high efficacy of pesticides when applied several times.

### Management of empty pesticide containers and expired pesticides

Empty pesticide containers and sachets were discarded indiscriminately after pesticide application (36%, *n* = 76/211). Some farmers discarded the empty containers in the trash (31.3%, *n* = 66/211), some burnt the containers (14.2%, *n* = 30/211), some buried them (7.6%, *n* = 16/211), and some recycled them for other purposes (10.9%, *n* = 23/211). A total of 27% (*n* = 57/211) of farmers reported that pesticides and fertilizers used often perished. Among those having expired pesticides and fertilizers, 70.1% (*n* = 148/211) indicated throwing them away, 15.2% (*n* = 32/211) buried them, 3.3% (*n* = 7/211) burned them when possible, and 2.4% (*n* = 5/211) said they returned the pesticide to the vendor or used them (3.8%, *n* = 8/211).

## Discussion

Regular surveillance of vector susceptibility to insecticides is crucial for the management of insecticide resistance, which greatly affects the control and elimination of malaria [29]. Two species, namely *An. gambiae* (s.s.) and *An. coluzzii*, are present in Yaoundé. These species play major roles in malaria transmission in Cameroon [8, 49–53]. Unplanned urbanisation and the practice of urban agriculture provide suitable breeding habitats for these species in Yaoundé [54–57]. A high insecticide resistance profile to different insecticide classes was detected, in conformity with previous studies [10, 11, 13, 50]. The evolution of insecticide resistance in the present study was consistent with studies conducted across Africa supporting the rapid expansion of insecticide resistance to various compounds [58–63]. Anopheline from Yaoundé also displayed resistance to high concentrations of permethrin and deltamethrin. Pyrethroids are the main compounds recommended for bednet impregnation [44]. The increasing tolerance of mosquitoes to high permethrin and deltamethrin doses is concerning and could jeopardize control efforts if nothing is done, so further attention is required. Several studies also suggested rapid expansion of pyrethroid resistance affecting all anopheline species including *An. gambiae* (s.s.), *An. coluzzii*, *An. arabiensis*, *An. funestus* and *Culex* species in the country [7, 50, 64–70].

High frequency of *kdr* allele 1014F was recorded. This allele had been reportedly involved in most cases of resistance to both pyrethroids and DDT [7, 10]. A low frequency of the *kdr* 1014S allele was recorded, supporting no major role for this allele. It is likely that metabolic base mechanisms are involved in mosquito resistance. The overexpression of several P450 detoxification genes such as *Cyp6M2*, *Cyp6P3* and *Cyp9K1* has been reported in mosquitoes from the city of Yaoundé [55, 64]. Concerning resistance to the carbamate bendiocarb, it could be induced by the presence of the mutation *ACE-1* and the overexpression of different detoxification genes [11, 64, 71]. Resistance to both carbamates and organophosphates is now expanding in malaria vectors across Africa [60, 62, 72–74].

Monitoring mosquito population susceptibility and resistance genes between 2017 and 2019 suggested high variability from one year to another. The evolution of insecticide resistance could be closely linked to the evolution of selective pressure induced by pesticide use in agriculture. From the survey conducted on pesticide usage, knowledge and management practices among vendors and farmers in the city of Yaoundé, it appeared that an increasing number of compounds are now used for controlling pests in agriculture. This same trend was recorded in other parts of the country [37, 75–77]. This study shows that more than 150 agricultural pesticides are sold on the market. The frequent usage of these compounds and their presence in breeding habitats might also contribute to the selection, development and spread of resistance in mosquitoes [78–81].

Many active ingredients were recorded in pesticides sold on the market by retailers. Studies conducted in Tanzania similarly identify a high number of formulations sold by retailers [82]. Different classes of insecticides including organophosphates, organochlorines, carbamates, pyrethroids and neonicotinoid acid were recorded in pesticides sold on the market. Some of the compounds such as neonicotinoids (imidacloprid, acetamiprid, thiamethoxam, thiacloprid and clothianidin) are new classes of insecticides that recently received approval for use in public health [83, 84]. The use of these compounds in agriculture could rapidly lead to insecticide resistance and affect the sustainability of new neonicotinoid base control tools. Neonicotinoids act by exerting neurotoxic effects via irreversible binding to insect nicotinic acetylcholine receptors [85]. Other insecticide classes are also used in both public health and agriculture and could select for insecticide resistance in mosquitoes. Several studies have emphasized the potential role of pesticides in the selection of insecticide resistance [15, 82, 86–89]. Glyphosate, atrazine, paraquat and dichloro-phenoxy acetic acid were common active ingredients in herbicides. These compound are considered to cause metabolic stress and can induce overexpression of genes involved in insecticide resistance [90–94]. Mixture of insecticides and fungicides was largely practiced by farmers. Other mixtures included insecticides + fungicides + nematicides + acaricides and insecticides + fungicides + herbicides. These mixtures could lead to the production of compounds highly toxic to plants and non-target organisms, harmful to the ecosystem, environment and farm operators [95–101]. Although most pesticides sold were on the list of pesticides approved for use in Cameroon [102], several formulations or versions of poor quality with the same brand were found in the market. The number of counterfeit agricultural products in Cameroon may be high because of poor check points along the boundaries with neighbouring countries [34, 37, 103, 104]. Some farmers admitted to applying up to six rounds of pesticides during the crop-growing stages. This extensive use as well as ineffective application of pesticides was also observed by many authors [37, 105–108] and is in accordance with the poor quality of pesticides sold on the market. Although all the retailers admitted to regularly providing advice to end-users, most of them had never received training on the use of these compounds. Retailers advise farmers on the choice of pesticides and the dosage based on their experience. Farmers on the other end did not have knowledge about crop pests, diseases, pesticide usage and management of pesticides. They mainly relied on information provided by pesticide retailers, other farmers and friends and sometimes from their personal work experience. Most of the farmers admitted never following the recommended dose; these findings are similar to those of studies conducted in other places [14, 17, 82, 100]. Pesticide application in farms varied according to season. There was a high usage of pesticides during the rainy season. Similar observations were made in the southern part of Ivory Coast, where a high utilization of insecticides and herbicides was noted during the rainy season [17]. In Tanzania, high utilization of pesticides and fungicides was recorded instead in the dry season by farmers cultivating rice and vegetables [82]. Studies conducted so far in the city of Yaoundé indicated moderate variability of susceptibility of *An. gambiae* (s.l.) to insecticides according to seasonal or temporal variations or type of breeding habitats [10, 50]. Unsafe storage and disposal of expired pesticides and empty pesticide containers were recorded, showing the need for more awareness among farmers and the community to promote best practices [105, 109–112].

In the light of the present study, it will be important for future studies to look for an association between exposure to agricultural pesticides and resistance selection in malaria vectors. Concerning usage practices, it is important to also interview key managers from relevant ministries to identify regulatory processes in place for the control, supply and selling of pesticides in the country. The study also sheds light on the evolution of insecticide resistance in malaria vectors and on putative factors which could exacerbate the spread of insecticide resistance, highlighting the need for increased collaboration between the agricultural and public health sectors for better management of insecticide resistance.

## Conclusions

The study confirmed the rapid evolution of insecticide resistance in *An. gambiae* (s.l.) populations in Yaoundé and the possible influence of pesticide use by farmers on the development of insecticide resistance. The study calls for increased action towards the population through education and sensitization campaigns to improve the use and management of pesticides and the environment. The study also stresses the need for concerted actions between actors from public health and agricultural sectors for the control and elimination of malaria.

## Supplementary Information

**Additional file 1: Table S1.** Survey sheet on pesticide vendors and farmers in the city of Yaoundé, Cameroon.

**Additional file 2: Table S2.** Trade names, types, active ingredients, WHO toxicity and chemical classes, and dose of pesticides used by farmers in Yaoundé, Cameroon.

## Data Availability

The datasets supporting the findings of this article are included within the published article and its additional files.

## Abbreviations

*ACE-1*: Acetylcholinesterase-1
CI: Confidence interval
CS: Capsule suspension
DDT: Dichlorodiphenyltrichloroethane
Delta: Deltamethrin
DNA: Deoxyribonucleic acid
EC: Emulsifiable concentrate
*GSTe2*: Glutathione S-transferase epsilon 2
IRS: Indoor residual spraying
KAP: Knowledge, attitudes and practices
*Kdr*: Knockdown resistance
LLINs: Long-lasting insecticidal nets
OCEAC: Organization for the Coordination of the fight against Endemic diseases in Central Africa
PBO: Piperonyl butoxide
PCR: Polymerase chain reaction
Perm: Permethrin
RR: Resistant homozygote
RS: Resistant heterozygotes
SC: Suspension concentrate
s.l.: Sensu lato
SL: Soluble concentrate
s.s.: Sensu stricto
SS: Susceptible homozygote
*VGSC*: Voltage-gated sodium channel
WHO: World Health Organization
WP: Wettable powders
