# Supplementary material for: Rapid evolution of insecticide resistance and patterns of pesticides usage in agriculture in the city of Yaoundé, Cameroon
Source: Parasit Vectors. 2022 Jun 2;15:186. doi: 10.1186/s13071-022-05321-8 (PMC9164381; doi:10.1186/s13071-022-05321-8)
Supplement: Supplementary file 1 — Additional file 1: Table S1. Survey sheet on pesticide vendors and farmers in the city of Yaoundé, Cameroon. [file 13071_2022_5321_MOESM1_ESM.docx]

ORGANISATION DE COORDINATION POUR LA LUTTE CONTRE LES ENDEMIES EN AFRIQUE CENTRALE


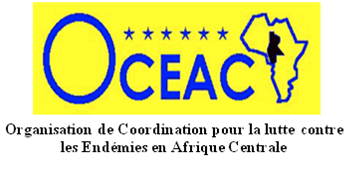


P.O. Box 288, Yaoundé, Cameroon

Tel: + 237 22232232

Fax: + 237 22230061

Web: http:// www.oceac.org

**Additional file 1: Table S1** Survey sheet for pesticide vendors and farmers in the city of Yaoundé Cameroon

| **I. SOCIO-DEMOGRAPHIC INFORMATION ON VENDORS OR FARMERS** **TAKING PART IN THE SURVEY** | | | |
| --- | --- | --- | --- |
| 1 | Date |  | |
| 2 | Investigator name |  | |
| 3 | Survey type | 1- VENDORS | |
|  |  | 2- FARMERS | |
| 4 | Site surveyed |  | |
| 5 | Participant Code |  | |
| 6 | Occupation |  | |
| 7 | Education level | 1- Primary school | |
|  |  | 2- Secondary school | |
|  |  | 3- University | |
|  |  | 4- No formal training | |
| 8 | Age (years) |  | |
| 9 | Gender | a- Male | |
|  |  | b- Female | |
| 10 | Land size | 1- <2000 m^2^ | |
|  |  | 2- 2000 m^2^-1 ha | |
|  |  | 3- >1 ha | |
| 11 | Crops cultivated |  | |
| **II. KNOWLEDGE AND PRACTICES REGARDING PESTICIDE VENDORS** | | | |
| 1 | What types of pesticides do you sell? |  | |
| 2 | Origin of pesticides sold | 1- Asia | |
|  |  | 2- Europe | |
|  |  | 3- Africa | |
|  |  | 4- America | |
| 2 | What is the purpose of using pesticides and fertilizers? | 1- Agriculture | |
|  |  | 2- Indoor spraying | |
| 4 | When do you sell the most pesticides and fertilizers? | 1- March-June (rainy season) | |
|  |  | 2- July-August (dry season) | |
|  |  | 3- Sept-Oct (rainy season) | |
|  |  | 4- Nov-February (dry season) | |
| 5 | Do you advice customers? | 1- Yes | a- Sometimes |
|  |  |  | b- Regularly |
|  |  | 2- No | |
| 6 | What kind of advice do you give to your customers? | 1- Choice of product | |
|  |  | 2- Dosage | |
|  |  | 3- Personal protection | |
| 7 | Do pesticides and fertilizers  often perish? | 1- Yes | |
|  |  | 2- No | |
| 8 | What do you do with expired  pesticides and fertilizers? | 1- Return to suppliers | |
|  |  | 2- Used on the field | |
|  |  | 3- Selling them | |
|  |  | 4- Throwing in the trash | |
| **III. KNOWLEDGE AND PRACTICES REGARDING PESTICIDE**  **USE BY FARMERS** | | | |
| 1 | Do you use pesticides and  synthetic fertilizers? |  | |
| 2 | Which type of pesticides do you used? |  | |
| 3 | Pests targeted |  | |
| 4 | Where did you learn how  to use pesticides? | 1- Advice | |
|  |  | 2- Training | |
|  |  | 3- Label | |
| 5 | Are you the one  making the dilutions? | 1- Yes | |
|  |  | 2- No | |
| 6 | On what basis do you  make the dilutions? | 1- Randomly | |
|  |  | 2- Instructions on label | |
|  |  | 3- Advice from suppliers and others | |
|  |  | 4- Training or seminar | |
| 7 | At what season do you use  pesticides and fertilizers? | 1- Dry season | |
|  |  | 2- All seasons | |
| 8 | Do you respect the recommended doses? | 1- Yes (standard doses) | |
|  |  | 2- No (high doses) | |
| 9 | Frequency of pesticides  application during plant  cultivation | 1- 1 time | |
|  |  | 2- Several times | |
| 10 | Are these pesticides  effective? | 1- Yes | |
|  |  | 2- No | |
| 11 | What do you do with the  empty containers? | 1- Burn | |
|  |  | 2- Bury | |
|  |  | 3- Throw in the trash | |
|  |  | 4- Discard indiscriminately | |
|  |  | 5- Keep for recycling | |
| 12 | Do pesticides and  fertilizers often perish? | 1- Yes | |
|  |  | 2- No | |
|  |  | 3- No answer | |
| 13 | What do you do with  expired pesticides and  fertilizers? | 1- Burn | |
|  |  | 2- Bury | |
|  |  | 3- Throw away | |
|  |  | 4- Use | |
|  |  | 5- Return to the suppliers | |
|  |  | 6- No answer | |
| 14 | Do you protect yourself when manipulating pesticides and fertilizers? | 1- Yes | |
|  |  | 2- No | |
