# Supplementary material for: Rapid evolution of insecticide resistance and patterns of pesticides usage in agriculture in the city of Yaoundé, Cameroon
Source: Parasit Vectors. 2022 Jun 2;15:186. doi: 10.1186/s13071-022-05321-8 (PMC9164381; doi:10.1186/s13071-022-05321-8)
Supplement: Supplementary file 2 — Additional file 2: Table S2. Trade names, types, active ingredients, WHO toxicity and chemical classes, and dose of pesticides used by farmers in Yaoundé, Cameroon. [file 13071_2022_5321_MOESM2_ESM.docx]

**Additional file 2: Table S2** Trade names, types, active ingredients, WHO toxicity and chemical classes, and dose of pesticides used by farmers in Yaoundé Cameroon

| **Trade Name** | **Pesticide type** | **Active ingredient (s)** | **WHO Toxic Classification** | **Chemical class** | **Dose** |
| --- | --- | --- | --- | --- | --- |
| Cofresh Gold 90 EC | Insecticide | 30 g per L imidacloprid, 60 g per L lambda-cyhalothrin | II | Neonicotinoid and pyrethroids | 0.5 to 0.75 L per ha i.e. 25 to 30 mL for a knapsack sprayer of 15 L water |
| Mamira Super 90EC | Insecticide | 30 g per L imidacloprid, 60 g per L lambda-cyhalothrin | II | Neonicotinoid and pyrethroids | 0.5 to 0.75 L per ha i.e. 25 to 30 mL for a knapsack sprayer of 15 L water |
| Kanon 90 EC | Insecticide | 30 g per L imidacloprid, 60 g per L lambda-cyhalothrin | II | Neonicotinoid and pyrethroids | 1 L per ha i.e. 40 mL for a 15 L sprayer and for 400 m^2^ |
| Callifan Super 40 EC | Insecticide | 20 g per L acetamiprid, 20 g per L bifenthrin | II | Nicotinoids and pyrethroids | 0.5 L per ha i.e. 25 mL for a knapsack sprayer of 15 L of water |
| Aceplant 40 EC | Insecticide | 40 g per L acetamiprid | Xn | Nicotinoids | 0.5 L per ha i.e. 25 mL for a knapsack sprayer of 15 L of water |
| Pyrinex Quick 256 SC | Insecticide | 250 g per L chlorpyrifos, 6 g per L deltamethrin | II | Organophospates and Pyrethroids | 0.3 L per ha i.e. 15 mL for a knapsack sprayer of 15 L of water |
| Alphacyca | Insecticide | 180 g per L alphamethrin, EC | II | Pyrethroids | 0.1 to 0.2 L per ha i.e. 5 to 10 mL sprayer and for 300 m^2^ |
| Cypercal^TM^ 50 EC | Insecticide | 50 g per L cypermethrin, EC | III | Pyrethroids | 0.6 to 0.9 L per ha i.e. 24 to 36 mL for a knapsack sprayer of 15 L of water |
| Plantima 30 SC | Insecticide | 30 g per L imidacloprid | II | Neonicotinoid | 1 L per ha i.e. 30 to 50 mL for a knapsack sprayer of 15 L of water |
| Cyplamdim Super | Insecticide | 15 g per L lambda-cyhalothrin, 20 g per L acetamiprid, EC | Xn | Pyrethroids and nicotinoids | 1 L per ha i.e. 50 mL for a knapsack sprayer of 15 L of water |
| Diomant 35 EC | Insecticide | 15 g per L lambda-cyhalothrin, 20 g per L acetamiprid, EC | II | Pyrethroids and nicotinoids | 1 L per ha i.e. 40 mL for a knapsack sprayer of 15 L of water |
| Pyriforce® | Insecticide | 600 g per L chlorpyrifos-Ethyl, EC | II | Organophosphates | 1 L per ha i.e. 37.5 mL for a knapsack sprayer of 15 L of water and for 20 sprayers per ha |
| Pyristor 600 EC | Insecticide | 600 g per L chlorpyrifos-Ethyl, EC | II | Organophosphates | 0.5 to 0.75 L per ha for a knapsack sprayer of 15 L water |
| K-optimal® | Insecticide | 15 g per L lambda-cyhalothrin, 20 g per L acetamiprid, EC | II | Pyrethroids and nicotinoids | 1 L per ha i.e. 40 mL for a 15 L sprayer and for 400 m^2^ |
| Onex Super | Insecticide | 20 g per L acetamiprid, 20 g per L cypermethrin | II | Nicotinoids and pyrethroids | 500 mL per ha mist blower i.e. 125 mL for a 10 L sprayer and for 2500 m^2^ |
| Galaxy 80EC | Insecticide | 40 g per L imidacloprid, 40 g per L lambda-cyhalothrin | II | Neonicotinoid and pyrethroids | 0.5 to 0.7 L per ha i.e. 25 mL for a knapsack sprayer of 15 L water |
| Grosplant 480 EC | Insecticide | 480g per L chlorpyrifos, EC | II | Organophosphates | 0.5 to 0.7 L per ha i.e. 25 mL for a knapsack sprayer of 15 L water |
| Epervier 220 EC | Insecticide | 20 g per L cypermethrin, 200 g per L chlorpyrifos-Ethyl, EC | II | Pyrethroids and organophosphates | 1 L per ha i.e. 50 mL for a knapsack sprayer of 15 L water |
| Pacha® 25 EC | Insecticide | 15 g per L lambda-cyhalothrin, 10 g per L acetamiprid, EC | II | Pyrethroids and nicotinoids | 300 L per ha i.e. 50 mL for a knapsack sprayer of 15 L of water |
| Gongfut 50EC | Insecticide | 1% imidacloprid, 4% cypermethrin, EC | II | Neonicotinoid and pyrethroids | 400 to 600 mL per ha sprayer |
| Cypercot | Insecticide | 100 g per L cypermethrin, EC | II | Pyrethroids | 20 to 25 sprayer per ha i.e. 20 mL for a knapsack sprayer of 16 L of water |
| Tamega R 25EC | Insecticide | 25 g per L deltamethrin, EC | II | Pyrethroids | 25 mL for a 15 L sprayer and for 300 m^2^ |
| Decis R 25EC | Insecticide | 25 g per L deltamethrin, EC | II | Pyrethroids | 0.5 L per ha |
| Antouka R 50 | Insecticide | 500 g per L pirimiphos methyl | II | Organophospates | 500 mL in 25 L of water and spray it on 500 m2 |
| Parastar 40 EC | Insecticide | 20 g per L imidacloprid, 20 g per L lambda-cyhalothrin | III | Neonicotinoid and pyrethroids | 0.5 L to 1 L per ha i.e. 25 to 40 mL for a knapsack sprayer of 15 L of water |
| Fixe 50 EC | Insecticide | 50 g per L fipronil, EC |  | Phenyl pyrazoles | 1 to 3 L per ha |
| Acarius R | Insecticide | 18 g per L abamectin, EC |  | Macrocyclic lactones | 25 to 50 mL within 15 L of water for a spray mixture of 300 L per ha |
| Abamex 018 EC | Insecticide | 18 g per L abamectin, EC |  | Macrocyclic lactones | 25 to 50 mL in 16 L knapsack sprayer |
| Fongicur R | Fungicide | 50 g per L hexaconazole, EC | II | Triazoles | 25 mL within 15 L of water for a spray mixture of 300 L per ha |
| Plantineb 80 WP R | Fungicide | 80% maneb, WP | II | Dithiocarbamate | 2 to 2.5 kg per ha in a mixture with water |
| Penncozeb 80 wp | Fungicide | 800 g per kg mancozeb, WP | III | Dithiocarbamate | 2 kg per ha i.e. 80 to 100 g per 16L knapsack sprayer |
| Persistent | Fungicide | chlorothalonil, 36% cymoxanil, WP |  | Organochlorines and acylalanine | 1 kg per ha |
| Terazeb 80 WP | Fungicide | 800 g per kg mancozeb, WP | III | Dithiocarbamate | 100 to 150 g for a knapsack sprayer of 15 L of water |
| MaxXzeb 80 WP | Fungicide | 800 g per kg mancozeb, WP | U | Dithiocarbamate | 2 kg per ha i.e. 60 to 80 g for a knapsack sprayer of 15 L |
| Grezeb 80WT | Fungicide | 800 g per kg mancozeb, WP | III | Dithiocarbamate | 2 kg per ha i.e. 100 g for a knapsack sprayer of 16 L |
| Banko R plus | Fungicide | 550 g per L chlorothalonil, 100 g per L carbendazim |  | Organochlorines and benzimidazole | 3 L per ha i.e. 125 mL for a sprayer of  15 L and for 400 m^2^ |
| Dekat-D R 720 SL | Herbicide | 720 g per L 2,4-D-dimethylamine salt |  | Aryloxyacides | 2.5 L per ha |
| Casse tout 480 SL | Herbicide | 480 g per L glyphosate | III | Amino-phosphonates | 75 mL per 16 sprayers and around 1.5 L per ha |
| Glyphader R 360 SL | Herbicide | 360 g per L glyphosate | U | Amino-phosphonates | 1.5 to 3 L per ha i.e. 60 to 120 mL for a knapsack sprayer of 16 L water and for 25 sprayers per ha |
| Glyfomax | Herbicide | 360 g per L glyphosate | U | Amino-phosphonates | 4 L per ha |
| Herbextra R | Herbicide | 720 g per L 2,4-D amine salt |  | Aryloxyacides | 40 to 80 mL in 16 L knapsack sprayer |
| Decassec | Herbicide | 720 g per L 2,4-D amine salt |  | Aryloxyacides | 1.5 L per ha |
| Aproxone 200 SL | Herbicide | 200 g per L paraquat |  | Pyridines | 3 to 5 L per ha |
| Bastion R Super | Nematicide, Acaricide | 5% oxamyl, GR |  | Carbamates | 60 kg per ha and for 21 days interval |
| Furadent Super 10G | Nematicide, Acaricide | 100 g oxamyl |  | Carbamates | 10 kg per ha i.e. 1 g per m^2^ |
| Caviar 48 EC | Herbicide | 665 g per L triclopyr butoxyethyl ester, 480 g per L triclopyr acid equivalent |  | Pyridines | 2 to 3 L per ha |
| CYPERCOT PLUS | Insecticide | 25% E.C. cypermethrin |  | Pyrethroids | 8 mL for a knapsack sprayer of 16 L and 20 to 25 sprayers per ha |
| BALLECOT 4.9 CS | Insecticide | 4.9% CS lambda-cyhalothrin |  | Pyrethroids | 250-500 g per mL in 400 to 600 L after 4 to 21 days |
| AGROPIK plus | Fungicide | 550 g per L chlorothalonil, 100 g per L carbendazim, SC | III | Organochlorines and benzimidazole | 50 mL in 15 L knapsack sprayer per ha |
| GLYCOL 41% SL | Herbicide | 480 g per L isopropylamine salt, equivalent to 360 g per L glyphosate acid |  | Aryloxyacides | 4 to 8 L per ha, i.e. sprayer: 1L for 50 L of water, atomizer: 1 L from 20 to 25 L of water |
| CYPERMULK 100 EC | Insecticide | 100 g per L cypermethrin, EC | II | Pyrethroids | 0.5 L per ha, i.e. 25 mL for a knapsack sprayer of 15 L or 50 mL for an atomizer |
| BOMEC R 18 EC | Nematicide, Acaricide | 18 g per L abamectin, EC |  | Macrocyclic lactones | 0.5 to 1.25 L per ha, i.e. 20 to 50 mL in a 15 L knapsack sprayer in order to spray around 400 sq.m. |
| ASSISTANT 720 SL | Herbicide | 720 g per L 2,4-D amine salt, SL |  | Aryloxyacides | 1 to 2 L per ha, i.e. 40 to 80 mL in 15 L knapsack sprayer |
| TROPIK 720 SC | Fungicide | 720 g per L chlorothalonil |  | Organochlorines | 1 to 5 L per ha |
| FIPROCOT 50 SC | Insecticide | 50 g per L fipronil, SC |  | Phenyl pyrazoles | 25 to 45 mL in 16 L sprayer and mix it, i.e. use 20 to 25 spray tanks for 1 ha |
| MAMIRA SUPER 90 EC | Insecticide | 30 g per L imidacloprid, 60 g per L lambda-cyhalothrin | II | Neonicotinoid and pyrethroids | 0.5 to 0.75 L per ha, i.e. 25 to 30 mL for a knapsack sprayer of 15 L water or 50 to 60 mL for an atomizer |
| CHLORCOT 480 EC | Insecticide | 480 g per L chlorpyrifos-Ethyl, EC |  | Organophosphates | 30 mL to 2 L in 15 L knapsack sprayer per ha |
| DINACACAO EC | Insecticide | 20 g per L imidacloprid, 20 g per L lambda-cyhalothrin | Xn | Neonicotinoid and pyrethroids | 1 L per ha, i.e. 50 mL in 15 L knapsack sprayer |
| DIURAX 800 SC | Herbicide | 800 g per L of diuron as CS | III | Substituted urea | 2 to 3 L per ha |
| TERAFORCE 600 EC | Insecticide | 600 g per L chlorpyrifos-Ethyl, EC |  | Organophosphates | 1 L per ha, i.e. 37.5 mL for a knapsack sprayer of 16 L and for 20 sprayers per ha |
| CYPERCAL TM 50 EC | Insecticide | 50 g per L cypermethrin, EC | III | Pyrethroids | 0.6 to 0.9 L per ha, i.e. 24 to 36 mL for a knapsack sprayer of 16 L of water |
| PYRIFORCE R | Insecticide | 600 g per L chlorpyrifos-Ethyl, EC |  | Organophosphates | 1 L to 1.5 L per ha, i.e. 37.5 to 60 mL for a knapsack sprayer of 16 L |
| CAIMAN R B | Insecticide | 50 g per kg emamectin benzoate, WG | II | Macrocyclic lactones | 50 g per kg for 1/2 ha, and 5 L of water for 10 g per knapsack sprayer of 16 L and for 420 m^2^ |
| CYPERPLANT 100 EC | Insecticide | 100 g per L cypermethrin, EC | II | Pyrethroids | 20 mL in 15 L of water |
| ACEPLANT 40 EC | Insecticide | 40 g per L acetamiprid | Xn | Neonicotinoid | 0.5 L per ha, i.e. 25 mL for a knapsack sprayer of 15 L water of water |
| Herbaclean 720 SL | Herbicide | 720 g per L 2,4-D amine salt, SL |  | Aryloxyacides | 1 to 2 L per ha, i.e. 40 to 80 mL for a knapsack sprayer of 16 L |
| Cleanfarm^TM^ 360 SL | Herbicide | 360 g per L glyphosate, 41% isopropylamine salt of glyphosate |  | Amino-phosphonates and aryloxyacides | 2 to 6 L per ha |
| MIRIDOR 45SC | Insecticide | 30 g per L thiamethoxam, 15 g per L lambda-cyhalothrin | II | Nicotinoids and pyrethroids | 1 L per ha, i.e. 45 to 50 mL per sprayer of 16 L |
| CAPSIDOR 50SC | Insecticide | 50 g per L fipronil, SC |  | Phenyl pyrazoles | 15 mL to 1 L per ha |
| Cleanfarm^TM^ 757 WSG | Herbicide | 360 g per L glyphosate, SL |  | Amino-phosphonates | 1 to 2 kg per ha, i.e. 300 to 400 L of water in a conventional spray |
| VYTAL 3G | Nematicide, Acaricide | 30 g per kg oxamyl | II | Carbamate | 35 to 50 kg per ha |
| LAMIDACOT 90 EC | Insecticide | 30 g per L imidacloprid, 60 g per L lambda-cyhalothrin | II | Neonicotinoids and pyrethroids | 0.5 to 0.75 L per ha, i.e. 25 to 30 mL for a knapsack sprayer of 15 L or 50 to 60 mL for an atomizer |
| CICAPSIDS 50SC | Insecticide | 50 g per L fipronil |  | Phenyl pyrazoles | 1 L per ha |
| SEPHINOR^TM^ 50 SC | Insecticide | 30 g per L imidacloprid, 20 g per L bifenthrin | II | Neonicotinoids and pyrethroids | 15 mL to 1 L per ha |
| LYNX | Insecticide | 15 g per L lambda-cyhalothrin, 20 g per L acetamiprid, EC | II | Pyrethroids and nicotinoids | 1 L per ha, i.e. 50 mL per sprayer of 15 L |
| SPAVOSATE R360 SL | Herbicide | 360 g per L glyphosate, SL |  | Amino-phosphonates | 1.5 to 2 L per ha when weeds are 30 to 40 cm high |
| PIRANHA 360 SL | Herbicide | 360 g per L glyphosate, SL | II | Amino-phosphonates | 4 to 8 L per ha |
| PARAFORCE | Insecticide | 600 g per L chlorpyrifos-Ethyl, EC |  | Organophosphates | 0.75 L per ha, i.e. 37.5 mL for a knapsack sprayer of 16 L |
| PLUSFORT 45 SC | Insecticide | 30 g per L thiamethoxam, 15 g per L lambda-cyhalothrin | II | Nicotinoids and pyrethroids | 45 to 50 mL for a knapsack sprayer of 15 L |
| GAMALIN 80 EC | Insecticide | 40 g per L imidacloprid, 40 g per L lambda-cyhalothrin | II | Neonicotinoids and pyrethroids | 0.5 L per ha, i.e. 25 mL per sprayer of 15 L |
| DOYEN62EC | Insecticide | 12 g per L emamectin benzoate, 50 g per L imidacloprid, EC |  | Macrocyclic lactones and neonicotinoids | 1 L per ha, i.e. 40 mL for a knapsack sprayer of 15 L in order to treat about 400 sq.m. |
| TOURBILLON SUPER 35 EC | Insecticide | 15 g per L lambda-cyhalothrin, 20 g per L acetamiprid, EC | II | Pyrethroids and nicotinoids | 1 L per ha, i.e. 40 mL for 15 L of water and for 400 m^2^ |
| EAGROWKIL | Insecticide | 480 g per L chlorpyrifos-Ethyl, EC |  | Organophosphates | 2 L per ha |
| SINOFORCE 600 EC | Insecticide | 600 g per L chlorpyrifos-Ethyl, EC | II | Organophosphates | 0.75 L per ha, i.e. 37.5 mL for a knapsack sprayer of 16 L or 75 mL for an atomizer |
| CIGOGNE R 360 EC | Insecticide | 360 g per L cypermethrin, EC |  | Pyrethroids | 100 mL per ha, i.e. 4 to 5 mL for 16 L knapsack sprayer |
| GROSPLANT 480 EC | Insecticide | 480 g per L chlorpyrifos-Ethyl, EC |  | Organophosphates | 15 to 20 mL in 15 L of water |
| DINASUPER 90EC | Insecticide | 30 g per L thiamethoxame, 60 g per L lambda cyhalothrin |  | Nicotinoids and pyrethroids | 360 to 480 mL per ha, i.e. 18 to 24 mL per sprayer of 15 L |
| BALEAR ^TM^ 720 SC | Fungicide | 720 g per L chlorothalonil, SC | III | Organochlorines | 2 to 3 L per ha |
| ELLAIME ^TM^ | Herbicide | 720 g per L 2,4-D amine salt, SC |  | Aryloxyacides | 1 L per ha, i.e. 400 mL to 1 L for 400 m^2^ per sprayer of 15 L |
| COTAMINE | Herbicide | 720 g per L 2,4-D amine salt, 28.0% inert ingredients, 60% acid equivalent |  | Aryloxyacides, inert ingredients and acid equivalent | 1 to 1.5 L per ha, i.e. 40 to 80 mL for a knapsack sprayer of 16 L |
| DIURALM 800 SC | Herbicide | 800 g per L of diuron, SC |  | Substituted urea | 0.75 to 2.25 L per ha |
| SUPER KILLER | Herbicide | 41% glyphosate, SL |  | Amino-phosphonates | 2 to 8 L per ha |
| COLIBRI | Insecticide | 30 g per L imidacloprid, SL | II | Neonicotinoids | 1L per ha, i.e. 30 to 80 mL per sprayer of 15 L or 80 to 120 mL for a mistblower of 10 L |
| IRON 30 SC | Insecticide | 30 g per L imidacloprid, SC |  | Neonicotinoids | 1L per ha or 50 mL per sprayer of 15 L or 250 mL for a 10 L mistblower (dose 1/4 per ha) |
| LADABA R 480 SL | Herbicide | 360 g per L glyphosate, SL |  | Amino-phosphonates | 1.5 to 3 L per ha, i.e. 75 to 150 mL for a knapsack sprayer of 16 L and for 25 sprayers per ha |
| QUICLEAR | Herbicide | 360 g per L glyphosate, SL |  | Amino-phosphonates | 2 to 8 L per ha |
| CALLIHERBE R 720 SL | Herbicide | 720 g per L 2,4-D amine salt, SL | III | Aryloxyacides | 3 L per ha |
| HERBALM 720 SL | Herbicide | 720 g per L 2,4-D dimethylamine salt, 600 g per L 2,4-D acid equivalent, SL |  | Aryloxyacides and acid equivalent | 1 to 2 L per ha |
| MALILO 100 EC | Herbicide | 180 g per L haloxyfop-R-methyl, EC |  | Pyridines | 225 mL within 1 sprayer and 4 sprayer per ha |
| GAWA 30 SC | Insecticide | 30 g per L imidacloprid, SC | II | Neonicotinoids | 1L per ha, i.e. 30 to 50 mL per sprayer of 15 L or 80 to 120 mL for a mistblower of 10 L |
| AZOX R 250 SC | Fungicide | 250 g per L azoxystrobin, SC |  | Strobilurin | 1 L per ha |
| Capsigro 50 SC | Insecticide | 50 g per L fipronil, SC |  | Phenyl pyrazoles | 600 mL per ha, i.e. 15 to 45 mL for per sprayer of 15 L |
| CYPERCAL ^TM^ 12 EC | Insecticide | 12 g per L cypermethrin, EC | III | Pyrethroids | 3 to 4 mL per ha or 120 to 160 mL per sprayer of 15 L |
| CYPALM 360 EC | Insecticide | 360 g per L cypermethrin, EC |  | Pyrethroids | 100 mL per ha or 50 mL in 5 L of water and apply the spray on 1/2 ha |
| TAPIR | Insecticide | 600 g per L chlorpyrifos-Ethyl, EC | II | Organophosphates | 750 mL per ha, i.e. 37.5 mL per sprayer of 15 L |
| CYPERMAX 100 EC | Insecticide | 100 g per L cypermethrin, EC |  | Pyrethroids | 0.5 L per ha, i.e. 25 mL for a knapsack sprayer of 15 L or 50 mL for an atomizer |
| TONER SUPER 45 SC | Insecticide | 30 g per L thiamethoxam, 15 g per L lambda-cyhalothrin |  | Nicotinoids and pyrethroids | 1 L per ha, i.e. 45 to 50 mL for a knapsack sprayer of 15 L |
| GREBTAC 100 EC | Insecticide | 100 g per L beta-cypermethrin, EC | II | Pyrethroids | 10 to 15 ml per ha |
| LAPLANTIM 40EC | Insecticide | 20 g per L imidacloprid, 20 g per L lambda-cyhalothrin | Xn | Neonicotinoids and pyrethroids | 1 L per ha, i.e. 50 mL in 15 L knapsack sprayer |
| GRECAPSIDE 46EC | Insecticide | 16 g per L acetamiprid, 30 g per L bifenthrin | II | Nicotinoids and pyrethroids | 25 mL for a knapsack sprayer of 15 L or 75 mL for a mistblower of 10 L water |
| PANLTIREL 220 EC | Insecticide | 20 g per L cypermethrin, EC |  | Pyrethroids | 1 L per ha, i.e. 50 mL for a knapsack sprayer of 15 L of water |
| STAROXONE 200 SL | Herbicide | 200 g per L paraquat, SL |  | Pyridines | 1 to 4 L per ha |
| CALLOXONE SUPER 200 SL | Herbicide | 200 g per L paraquat, SL | Ib | Pyridines | 2 to 4 L per ha |
| RAFALE | Herbicide | 200 g per L paraquat, SL |  | Pyridines | 2 to 4 L per ha |
| GRASSKILLER 200 SL | Herbicide | 200 g per L paraquat, SL | II | Pyridines | 3 to 6 L per ha, i.e. 150 to 300 mL per sprayer |
| NICOMAIS R 40 SC | Herbicide | 40 g per L nicosulfuron, SC |  | Sulfonylurea | 1 L per ha |
| ULTRAMAIS R | Herbicide | 4.0 % nicosulfuron, 6.0 % fluroxypy | U | Sulfonylurea and auxine | 1 L per ha |
| CYPALM 200 E | Insecticide | 200 g per L cypermethrin, EC | II | Pyrethroids | 10 mL for a knapsack sprayer of 15 L |
| AFNICOT | Herbicide | 40 g per L nicosulfuron OD |  | Sulfonylurea | 1 to 1.5 L per ha |
| SINURON 800 SC | Herbicide | 800 g per L of diuron, SC | III | Substituted urea | 2 to 3 L per ha, i.e. 100 to 150 mL for a knapsack sprayer of 15 L |
| STAROMIL R 72WP | Fungicide | 12 g per kg dimethomorph, 600 g per kg copper oxide | III | Morpholine and metal oxide | 50 g for one 15 L sprayer |
| RIDOMIL GOLD R | Fungicide | 60 g per kg of mefenoxam, 600 g per kg of copper in form of cuprous oxide | III | Acylalanine and metal oxide | 50 g for one 15 L sprayer |
| CacaoMil 72 WP | Fungicide | 120 g per kg metalaxyl-M, 600 g per kg cuprous oxide |  | Acylalanine and metal oxide | 50 g for a knapsack sprayer of 15 L |
| MONCHAMP 720 WP | Fungicide | 120 g per kg cymoxanil, 600 g per kg mancozeb | III | Acylalanine and dithiocarbamate | 50 g into a knapsack sprayer tank of 15 L |
| BLACK OUT R | Fungicide | 60 g per kg metalaxyl-M, 600 g per kg cuprous oxide, WP |  | Acylalanine and metal oxide | 50 g into a knapsack sprayer of 15 L |
| SONAMIL 720 WP | Fungicide | 120 g per kg metalaxyl-M, 600 g per kg cuprous oxide | III | Acylalanine and metal oxide | 25 g in a knapsack sprayer of 15 L |
| PLANTOMIL SUPER | Fungicide | 60 g per kg metalaxyl-M, 600 g per kg cuprous oxide |  | Acylalanine and metal oxide | 5 g in a knapsack sprayer of 15 L |
| MANCOSAP | Fungicide | 800 g per kg mancozeb, WP | III | Dithiocarbamate | 100 to 150 g for a knapsack sprayer of 15 L |
| CLEANOMIL GOLD | Fungicide | 60 g per kg metalaxyl-M, 600 g per kg cuprous oxide | II | Acylalanine and metal oxide | 50 g for one knapsack sprayer of 15 L |
| FONGIPRO R | Fungicide | 120 g per kg cymoxanil, 700 g per kg oxychloride |  | Acylalanine and oxime + inorganic | 50 g in one sprayer for 20 knapsack sprayer per ha |
| FUGIFORCE 66 WP | Fungicide | 60 g per kg metalaxyl-M, 600 g per kg cuprous oxide, WP | III | Acylalanine and metal oxide | 50 g for one sprayer |
| METTALL-O+ | Fungicide | 120 g per kg dimethomorph, 600 g per kg cupper oxide | III | Morpholine and metal oxide | 50 g for 15 L of water |
| KOBIZEB 80 WP | Fungicide | 800 g per kg mancozeb, WP | III | Dithiocarbamate | 100 to 150 g for a knapsack sprayer of 15 L of water |
| MANCOSTAR 80WP | Fungicide | 800 g per kg mancozeb, WP | III | Dithiocarbamate | 40 to 100 g for a knapsack sprayer of 15 L |
| IVORY 80 WP | Fungicide | 800 g per kg mancozeb, WP | III | Dithiocarbamate | 40 to 100 g for a knapsack sprayer of 15 L |
| GREFONSEC COMPLEX 210 WP | Fungicide | 50 g per kg carbendazim, 120 g per kg sulfur, 20 g per kg imidacloprid | III | Benzimidazole, sulfur and neonicotinoids | 50 to 75 g for a knapsack sprayer of 16 L |
| CAPITAIN 72WP | Fungicide | 120 g per kg dimethomorph, 600 g per kg copper oxychloride | III | Morpholine and metal oxide | 50 per knapsack sprayer of 15 L of water |
| QUANTUM ROK TM | Fungicide | 60 g per kg dimethomorph, 140 g per kg copper hydroxide | III | Morpholine and hydroxide compound | 50 g for one knapsack sprayer of 15 L |
| KOBIOCHAMP R 72% WP | Fungicide | 80 g per kg mefenoxam, 640 g per kg mancozeb, WP | III | Acylalanine and dithiocarbamate | 50 g into a knapsack sprayer of 15 L |
| FUNGIOFF SUPPER 660 WP | Fungicide | 60 g per kg mefenoxam, 600 g per kg copper oxide | III | Acylalanine and metal oxide | 50 g for 15 L of water |
| OK MIL R Unik R | Fungicide | 60 g per kg metalaxyl-M, 600 g per kg cuprous oxide, WP | III | Acylalanine and metal oxide | 1 kg per ha, i.e. 50 g per 15 L knapsack sprayer per 500 m^2^ and 20 knapsack sprayer per ha |
| METRO STAR R | Fungicide | 150 g per kg thiophonate-Methyl, 200 g per kg copper oxychloride, 150 g per kg sulfur | III | Benzimidazole and metal oxide | 50 g in a knapsack sprayer of 16 L |
| CALLOMIL Super 66 WP | Fungicide | 60 g per kg metalaxyl-M, 600 g per kg cuprous oxide, WP | III | Acylalanine and metal oxide | 50 per knapsack sprayer of 15 L |
| ROMIL 66 WP | Fungicide | 60 g per kg mefenoxam, 600 g per kg copper oxide, WP | III | Acylalanine and metal oxide | 1 kg per ha, i.e. 50 g bag per 15 L sprayer or 250 g per atomizer |
| NORDOX 75 WG | Fungicide | 86% cuprous oxide, 14% inert ingredients, 75% metallic copper equivalent |  | Metal oxide and inert ingredients | 40 g net dosage for one sprayer of 15 L |
| ACTION R 80DF | Herbicide | 800 g per L of diuron, WG | III | Substituted urea | 0.675 to 180 g per 1/4 ha |
| BAOBAB 80 WP | Fungicide | 800 g per kg mancozeb, WP | III | Dithiocarbamate | 100 g per 15 L of water every 7 to 10 days |
| ROZEB 80 WP | Fungicide | 800 g per kg mancozeb, WP |  | Dithiocarbamate | 2 kg per ha or 100 g per 16 L sprayer |
| SINOMAIS 79 WG | Herbicide | 750 g of atrazine, 40 g of nicosulfuron | U | Dinitroanilines and sulfonylurea | 1 kg per ha, i.e. 50 g per 15 L sprayer |
| RAPID-ATTACK 344SE | Insecticide | 144 g per L of cypermethrin and 200 g per L of imidacloprid | II | Pyrethroids and neonicotinoids | 0.5 to 0.75 L per ha i.e. 25 to 30 mL for a knapsack sprayer of 15 L water |

EC, emulsifiable concentrate; SC, suspension concentrate; SL, soluble concentrate; WP, wettable powders; CS, capsule suspension. FAO/WHO classification: Class Ib, highly hazardous; class II, moderately hazardous; class III, slightly hazardous; class Xn, harmful; class U, unlikely to present acute hazard in normal use
